# Fabrication of Pore-Selective Metal-Nanoparticle-Functionalized Honeycomb Films via the Breath Figure Accompanied by In Situ Reduction

**DOI:** 10.3390/polym13030316

**Published:** 2021-01-20

**Authors:** Yongjiang Li, Xiaoyan Ma, Jingyu Ma, Zongwu Zhang, Zhaoqi Niu, Fang Chen

**Affiliations:** School of Chemistry and Chemical Engineering, Northwestern Polytechnical University, Xi’an 710129, China; huorekaluli@163.com (Y.L.); 995696861@mail.nwpu.edu.cn (J.M.); 740634019@mail.nwpu.edu.cn (Z.Z.); niuzhaoqi@mail.nwpu.edu.cn (Z.N.); chenfang820811@nwpu.edu.cn (F.C.)

**Keywords:** honeycomb films, POSS-based star-shaped polymers, pore-selective functionalization, metal nanoparticles, breath figure

## Abstract

Honeycomb films pore-filled with metal (Au, Ag, and Cu) nanoparticles were successfully prepared by combining the breath figure method and an in situ reduction reaction. First, a polyhedral oligomeric silsesquioxane (POSS)-based star-shaped polymer solution containing metal salt was cast under humid conditions for the formation of honeycomb films pore-filled with metal salt through the breath figure method. The morphology of the honeycomb films was mainly affected by the polymer molecular structure and the metal salt. Interestingly, the promoting effect of the metal salt in the breath figure process was also observed. Then, honeycomb films pore-filled with metal nanoparticles were obtained by in situ reduction of the honeycomb films pore-filled with metal salt using NaBH_4_. Notably, the metal nanoparticles can be selectively functionalized in the pores or on the surface of the honeycomb films by controlling the concentration of the NaBH_4_. Metal-nanoparticle-functionalized honeycomb films can prospectively be used in catalysis, flexible electrodes, surface-enhanced Raman spectroscopy (SERS), and wettability patterned surfaces.

## 1. Introduction

Honeycomb films have attracted considerable interest due to their numerous potential applications in the fields of size-selective separations [1], superhydrophobic surfaces [2], catalysis sensors [3], and so on. Although there are many methods for preparing porous films with different pore sizes, shapes, and morphologies, the breath figure (BF) method is a simple and fast method that uses condensed water droplet arrays as dynamic templates [4,5,6,7,8].

Functionalized honeycomb films have extensive and particular applications compared with pristine un-functionalized honeycomb films. The incorporation of inorganic components into polymeric honeycomb films enables synergistic coupling of multiple functionalities of each component. Because metal-nanoparticle-functionalized honeycomb films can be used as porous electrodes [3], water correctors [9,10], and surface-enhanced Raman spectroscopy (SERS) substrates [11], there has been much attention focused on preparing metal-nanoparticle-functionalized honeycomb films. Generally, the main approaches for the metal modifications of honeycomb films are performed after the BF process [12,13]. Some techniques have been developed for the metal modification of honeycomb films, such as chemical deposition [14], electrodeposition [11,15], electroless plating [16,17], vapor deposition [18], and physical adsorption [19]. Although these techniques were successfully applied in the fabrication of metallized honeycomb films, they involve elaborate procedures, post-modifications, and/or sophisticated instrumentation. Meanwhile, the superhydrophobicity of honeycomb films increases the difficulty of modifications. Therefore, other studies have illustrated the use of mixed solutions of polymers and metal nanoparticles to prepare metal-nanoparticle-functionalized films via the BF method, in which the metal nanoparticles may self-assemble at the polymer solution/water droplet interface and are left in the polymeric pore after the evaporation of the water droplets [20,21,22,23,24,25]. Recently, by adding metal salt precursors into a polymer solution, metal-oxide-nanoparticle-functionalized honeycomb films were obtained using the breath figure method accompanied by an in situ chemical reaction. Due to the good dispersibility or solubility in the polymer solution of their precursors, films functionalized with metal oxide nanoparticles, including TiO_2_ [26,27], ZnS [28], SnO_2_ [29], and SnS [30], were obtained by subsequently hydrolyzing or mineralizing the as-prepared films.

Furthermore, the addition of precursors (metal salt) could affect the morphology of the honeycomb films. For instance, Olga Naboka et al. reported the addition of CoCl_2_ to cellulose acetate (CA)/acetone to obtain CA/CoCl_2_ honeycomb films using the breath figure method [31]. They found that CoCl_2_ could promote the formation of honeycomb-patterned CA films. Hsu et al. found that LiCl addition significantly improved film porosity, and that the micro-pores appeared to be networked [32]. Moreover, the addition of metal salt into a substrate also affects the morphology of films. Sajjad developed an approach to preparing a polymer–Ag honeycomb film by casting a polymer solution on an aqueous silver nitrate solution [33]. They found that the metal-complexation-induced phase separation approach allowed the formation of a well-defined honeycomb film. Similarly, Do prepared asymmetrically functionalized poly (ε-caprolactone)/polypyrrole/silver honeycomb films by casting a polymer solution on a reactive ice substrate (containing AgNO_3_) [34]. Those studies have clearly demonstrated that metal salt has significant effects on the morphology of honeycomb films.

In the present work, the pore-selective filling of metal (Au, Ag, and Cu) nanoparticles in honeycomb films was prepared by combining the breath figure method and an in situ reduction reaction. Four polyhedral oligomeric silsesquioxane (POSS)-based star-shaped polymers mixed with metal salt were selected for analyzing the effects of polymer molecular structure and metal salt on the morphology of honeycomb films during the BF process. Then, honeycomb films pore-filled with metal nanoparticles were obtained by reducing the honeycomb films pore-filled with metal salt in situ using NaBH_4_. This work provides a new and facile approach to preparing honeycomb films in which the pores are selectively filled with metal nanoparticles.

## 2. Materials and Methods

### 2.1. Materials

**Figure 1:**
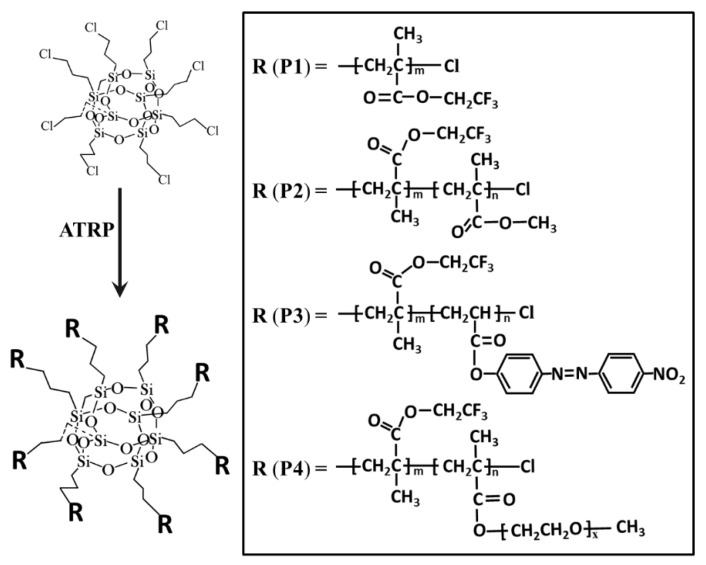
The chemical structure of the polyhedral oligomeric silsesquioxane (POSS)-based star-shaped polymers.

The four POSS-based star-shaped polymers used in the present study were synthesized previously by our laboratory [35,36], and their chemical structures and characteristics are listed in Figure 1 and Table 1. All reagents were of analytical grade and were used as received without further treatment. Water used in all of the experiments was deionized and ultrafiltrated to 18.2 MΩ.

### 2.2. Preparation of Honeycomb Films Pore-Filled with Metal Salt

The polymer solution was prepared by dissolving 80 mg of polymer in 1 mL of tetrahydrofuran (THF). The metal salt (including HAuCl_4_, AgNO_3_, CuCl_2_, FeCl_3_, and LiCl) aqueous solution (200 mg/mL) was then added into the polymer solution in different contents (wt%). The static breath figure method was employed to fabricate honeycomb films. The solution of the polymer mixed with metal salt was directly cast onto glass substrates under humid conditions (>80% relative humidity). During the solvent evaporation, the transparent solution turned turbid rapidly, and water droplets condensed at the air/solution interface. After complete evaporation of the solvent and water, the honeycomb films pore-filled with metal salt were prepared. For comparison, polymer films without metal salt were also fabricated under similar conditions.

### 2.3. Preparation of Metal-Nanoparticle-Functionalized Honeycomb Films

**Figure 2:**
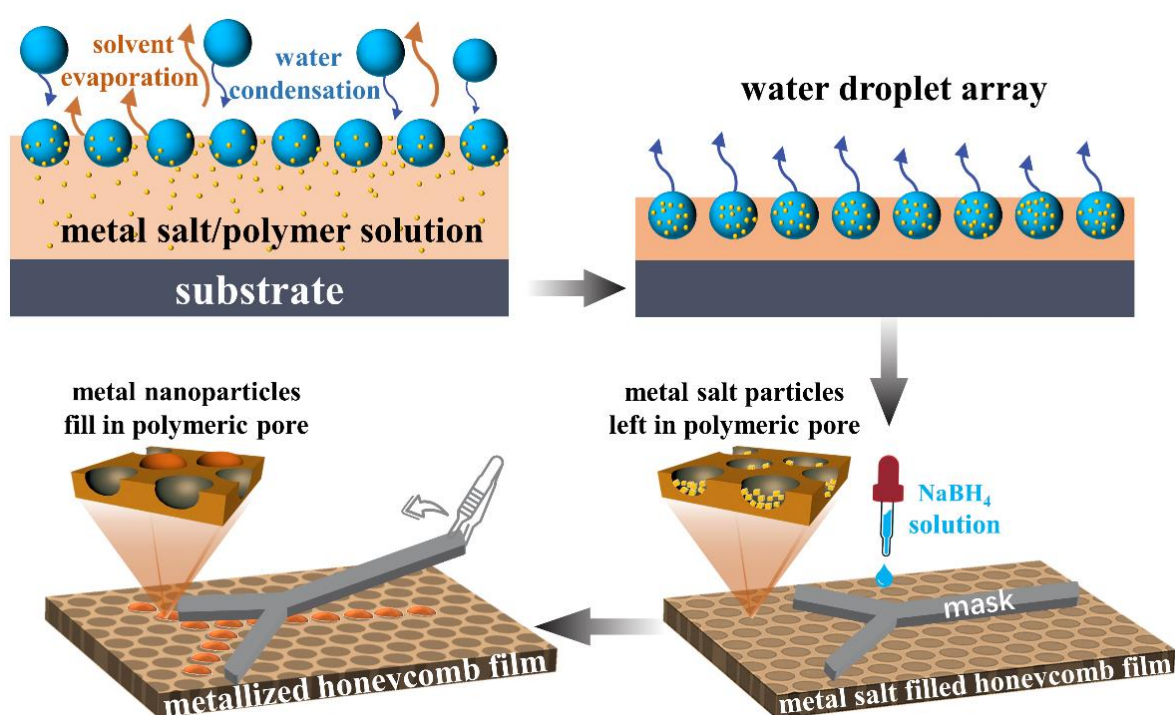
The schematic representation showing the fabrication of the metal-nanoparticle-functionalized honeycomb films.

The metal-nanoparticle-functionalized honeycomb films were obtained by reducing the metal-salt-filled (including HAuCl_4_, AgNO_3_, and CuCl_2_) honeycomb films using NaBH_4_. In addition, differently surface-patterned hybrid polymer–metal films were obtained by reducing the metal salt using the patterned masks. The patterned masks were prepared by cutting filter paper into different patterns. To be specific, a patterned mask was superimposed on the metal-salt-filled film, and then the NaBH_4_ aqueous solution was dropped on the mask. The honeycomb films pore-filled with metal salt were made to react with the NaBH_4_ aqueous solution for 1 h. Then, the mask was gently removed. The film was washed several times with water to remove the unreacted reagents, and was dried under vacuum at room temperature. Only the mask-patterned region was metallized. Different results were obtained at lower (0.05 mol/L) and higher (0.1 mol/L) concentrations of NaBH_4_. The schematic representation of the metal-nanoparticle-functionalized honeycomb film fabrication process is shown in Figure 2.

### 2.4. Characterization

Scanning electron microscopy (SEM) images were obtained by a VEGA 3 LMH (TESCAN, Brno, Czech Republic) with an accelerating voltage of 20 kV. The elemental composition distribution was determined using a scanning electron microscope equipped with energy dispersive spectrometry (EDS) (INCA X-ACT, Oxford Instruments, Abingdon, UK). Fourier-transform infrared (FTIR) spectra were recorded with a Bruker Tensor 27 spectrometer (Bruker, Billerica, MA, USA) at a resolution of 2 cm^−1^. The surface chemical analysis was performed with X-ray photo-electron spectroscopy (XPS, Kratos Analytical, Manchester, UK). The apparent contact angle was measured with the pendent drop method (JC2000D4 Powereach Tensionmeter, Shanghai Zhongchen Digital Technology Equipment Co., Ltd., Shanghai, China).

## 3. Results and Discussion

### 3.1. Effects on the Morphology of Honeycomb Films

**Figure 3:**
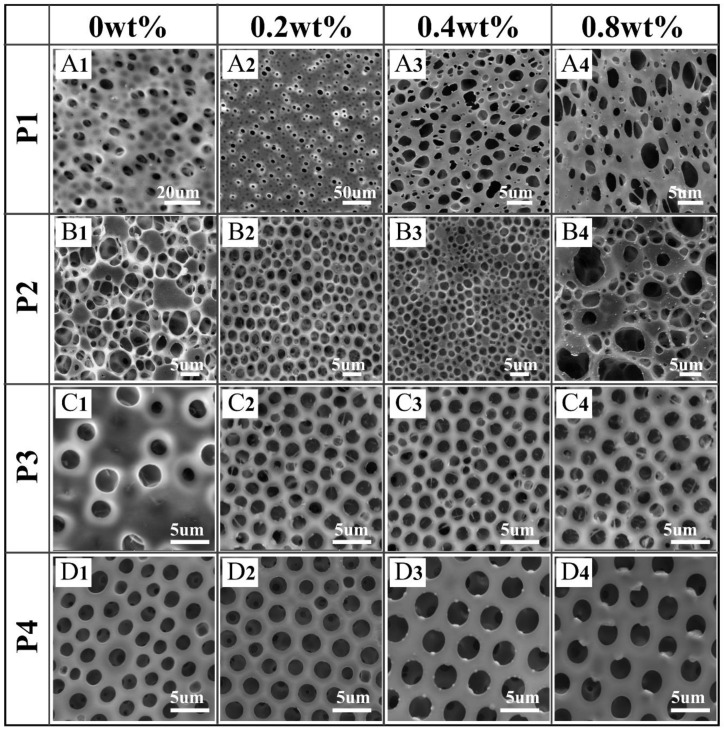
Scanning electron microscopy (SEM) images of honeycomb films (**A_1_**–**D_4_**) prepared using four POSS-based star-shaped polymer/tetrahydrofuran (THF) solutions with different contents of CuCl_2_.

Based on the mechanism of BF formation, nonpolar and highly volatile solvents are suitable for the BF process. As depicted in our previous contribution [36], the honeycomb films were obtained from polymers/CHCl_3_. However, in order to add the aqueous metal salt to the film-forming solution, THF was chosen as a solvent due to its water miscibility. As expected, films with disordered pores were obtained when the solvent was THF, as shown in Figure 3A_1_,B_1_,C_1,_D_1_. Interestingly, the addition of metal salt into different POSS-based star-shaped polymer/THF solutions has obviously different effects on the regularity and shape of the pores.

#### 3.1.1. Effect of Polymer Molecular Structures on the Morphology of Honeycomb Films

As shown in Figure 3A_1_,B_1_,C_1_,D_1_, films with disordered pores were obtained when the solvent was THF without CuCl_2_ because the droplets would coalesce and even mix with the polymer/THF solutions. Due to its good amphiphilic property, the film prepared with P4 was the most regular in the absence of CuCl_2_ (Figure 3D_1_).

**Figure 4:**
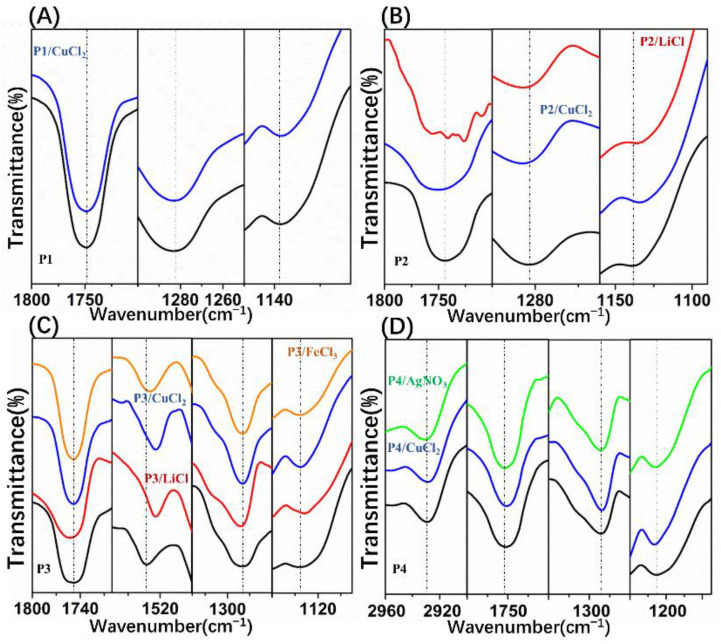
Fourier-transform infrared (FTIR) spectra for polymer/salt films: (**A**) pure P1 and P1/CuCl_2_ films; (**B**) pure P2, P2/LiCl, and P2/CuCl_2_ films; (**C**) pure P3, P3/LiCl, P3/CuCl_2_, and P3/FeCl_3_ films; (**D**) pure P4, P4/CuCl_2_, and P4/AgNO_3_ films.

Interestingly, relatively regular pores were obtained by addition of CuCl_2_ to the POSS-based star-shaped polymer solution, except for P1. Due to the superhydrophobicity of P1, it was difficult to capture CuCl_2_ to form polymer–Me^n+^ complexes that could stabilize the condensed water droplets. In the P1/CuCl_2_ FTIR spectra, the ester group band was unchanged compared to the spectrum of pure P1, which indicates that P1–Me^n+^ complexes were not formed (Figure 4A). Moreover, if the polymer solution is very hydrophobic, the nucleation of the water droplets will be suppressed. As a result, films with irregular pore arrays were obtained using P1. On the other hand, CuCl_2_ can induce the formation of honeycomb P2 films with relatively regular pores at 0.2 and 0.4 wt% content (Figure 3B_2_,B_3_), while disordered porous structures were obtained at 0.8 wt% content (Figure 3B_4_). Due to the poly(methyl methacrylate) (PMMA) structures, P2 is more hydrophilic than P1. Therefore, P2 can capture CuCl_2_ to form polymer–Me^n+^ complexes, which are beneficial for the stability of water droplets during the BF process. The main adsorption bands of the FTIR spectra of pure P2 are assigned to the stretching vibration of C=O from ester groups (1743 cm^−1^), as well as the symmetric and asymmetric stretching vibrations of C-O-C (1282 cm^−1^, 1139 cm^−1^). In the P2/CuCl_2_ FTIR spectra (Figure 4B), the stretching vibration of C=O and the symmetric and asymmetric stretching vibrations of C-O-C shifted to 1749,1286, and 1137 cm^−1^, respectively. These changes were due to the metal–ligand interactions between P2 and CuCl_2_.

Additionally, CuCl_2_ can induce the formation of honeycomb P3 and P4 films with regular pores at 0.2, 0.4, and 0.8 wt% content due to their amphiphilic property. In fact, the existence of polar groups in the amphiphilic copolymers, which could be associated with metal ions, played important roles in BF processes. In the P3/CuCl_2_ FTIR spectra (Figure 4C), the ester group band was unchanged compared to the spectrum of pure P3. The stretching vibration of Ar-NO_2_ was shifted toward a lower frequency (1521 cm^−1^) compared to the spectrum of pure P3. In the P4/CuCl_2_ FTIR spectra (Figure 4D), the stretching vibrations of -CH2- (from polyethylene glycol (PEG)) and C=O were shifted toward a lower frequency (2927 cm^−1^, 1751 cm^−1^) and the symmetric stretching vibration of C-O-C was shifted toward a higher frequency (1132 cm^−1^) compared to the spectrum of pure P4. In the P4/AgNO_3_ FTIR spectra (Figure 4D), the stretching vibration of -CH_2_- (from PEG) and symmetric stretching vibration of C-O-C were shifted toward higher frequencies (2931 cm^−1^, 1132 cm^−1^) compared to the spectrum of pure P4. Shifts in the polymer/CuCl_2_ FTIR spectra indicated the metal–ligand interactions between them.

Although P2 and P3 can capture CuCl_2_ to form polymer–Me^n+^ complexes, films with highly regular pores arrays were obtained by P4. This indicates that the amphiphilic property of polymers plays an important role in BF processes. Hence, P4 was chosen for the fabrication of pore-selective metal-nanoparticle-functionalized honeycomb films, and P2 and P3 were chosen to study the effect of metal salt on the morphology of the honeycomb films.

#### 3.1.2. Effect of Metal Salt Type and Content on the Morphology of Honeycomb Films

The addition of LiCl, CuCl_2_, or FeCl_3_ aqueous solution into the P3/THF solution with different contents was chosen to investigate the effect of the metal salt type and content on the surface morphology of the films. For comparison, the corresponding amounts of pure water were added into the polymer solution to fabricate the films.

**Figure 5:**
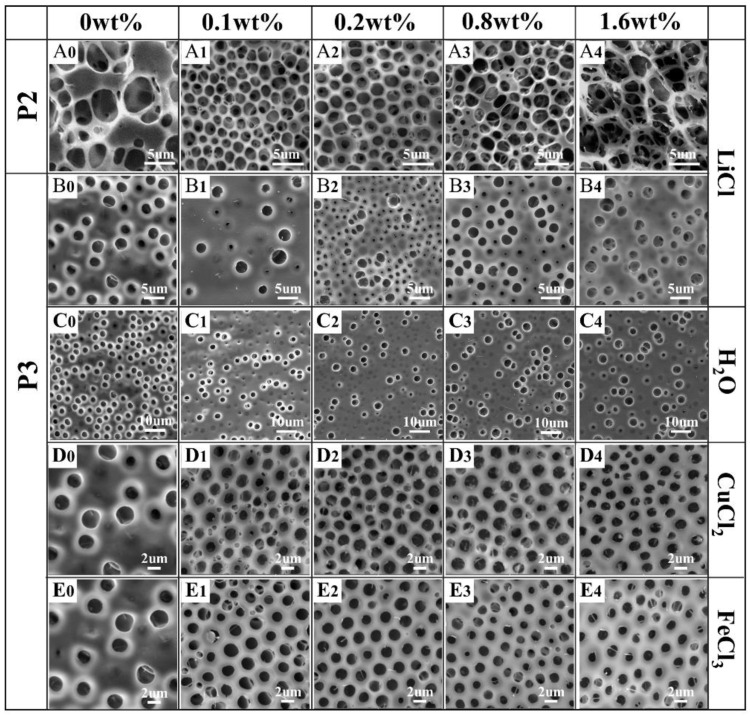
SEM images of honeycomb films prepared using P2 (**A_0_**–**A_4_**) and P3 (**B_0_**–**E_4_**) with different metal salt types and contents.

As expected, the addition of pure water into the P3/THF solution was unable to form regular patterns (Figure 5C_1_–C_4_). In the P3/LiCl FTIR spectra (Figure 4C), the stretching vibration of C=O and asymmetric stretching vibration of C-O-C were shifted toward higher frequencies (1753 cm^−1^, 1284 cm^−1^) and stretching vibration of Ar-NO_2_ and symmetric stretching vibration of C-O-C were shifted toward lower frequencies (1521 cm^−1^, 1132 cm^−1^) compared to the spectrum of pure P3. Although the shifts in the P3/LiCl FTIR spectra indicate metal–ligand interactions between P3 and LiCl, films with irregular pores were obtained using P3/LiCl (Figure 5B_1_–B_4_). On the other hand, films with relatively regular pores were obtained using P2/LiCl (Figure 5A_1_–A_3_). In the P2/LiCl FTIR spectra (Figure 4B), the asymmetric stretching vibration of C-O-C was shifted toward a higher frequency (1286 cm^−1^) and the stretching vibration of C=O and symmetric stretching vibration of C-O-C were shifted toward lower frequencies (1741 cm^−1^, 1134 cm^−1^) compared to the spectrum of pure P2. Presumably, due to their poor flexibility, it is difficult to form P3–Li^+^ complexes at the water–solution interface to stabilize the condensed water droplets. Fortunately, honeycomb films with relatively regular pores were successfully obtained using P3/CuCl_2_ and P3/FeCl_3_ (Figure 5D_1_–D_4_,E_1_–E_4_). They had similar effects on the regularity of the pores due to the similar metal–ligand interactions with P3. In the P3/FeCl_3_ FTIR spectra (Figure 4C), the ester group band was unchanged compared to the spectrum of pure P3. The stretching vibration of Ar-NO_2_ was shifted toward a lower frequency (1523 cm^−1^) compared to the spectrum of pure P3. Together, these observations indicated that the polymers tended to coordinate with transition metal ions to form polymer–Me^n+^ complexes at the water–solution interface to stabilize the condensed water droplets.

In addition to the above metal salt types, the morphology of pores can be affected by metal salt content. For instance, P2/CuCl_2_ films with relatively regular pores were obtained at 0.2 and 0.4 wt% content of CuCl_2_, while disordered porous structures were obtained at 0.8 wt% content of CuCl_2_ (Figure 3B_2_–B_4_), because they surpass the ability of the polymer–Me^n+^ complexes to stabilize the condensed water droplets. However, a higher content of CuCl_2_ or FeCl_3_ in P3 will lead to smaller pores on the films. The average pore size of P3/CuCl_2_ films decreased from 1.67 to 1.44 µm with the increase of CuCl_2_ content from 0.2 to 1.6 wt% (Figure 5D_2_,D_4_). Similarly, the average pore size of P3/FeCl_3_ films decreased from 1.68 to 1.45 µm with the increase in FeCl_3_ content from 0.2 to 1.6 wt% (Figure 5E_2_,E_4_).

### 3.2. Proposed Mechanism of the Effects on the BF Process

Amphiphilic block copolymers composed of hydrophilic and hydrophobic blocks are widely used in the fabrication of honeycomb films because they can assemble at the water–solution interface to stabilize the condensed water droplets [37]. The polar groups in the polymers can capture metal ions to form polymer–Me^n+^ complexes. Thus, the complexes become amphiphilic, with hydrophilic ionic structures and a hydrophobic core. As a consequence, the formation of polymer–Me^n+^ complexes is beneficial for the stability of water droplets during the BF process. The expected possible mechanism for the effects of polymer molecular structure and metal salt on the BF process can be explained as follows.

**Figure 6:**
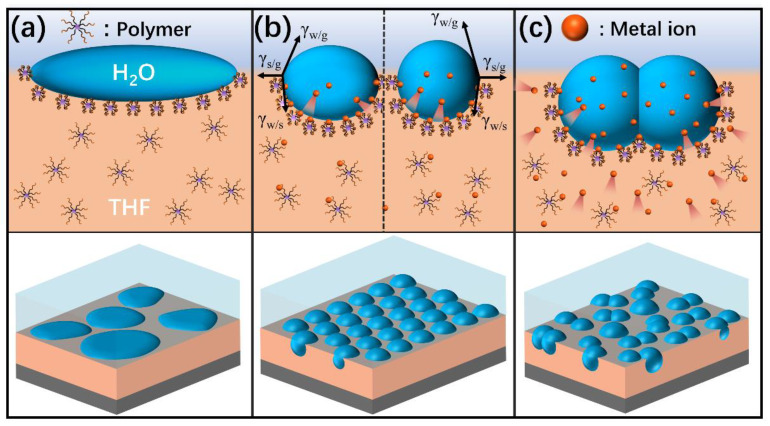
Schematic illustration of water droplets stabilized at the interface during breath figure formation: (**a**) polymer solution without metal salt; (**b**) addition of appropriate content of metal salt into the polymer solution; (**c**) addition of excess content of metal salt into the polymer solution.

First, films with disordered pores were obtained when the solvent was THF because the droplets would coalesce and even mix with polymers/THF due to the water miscibility of THF (Figure 6a). Among them, the films prepared using P4 were the most regular in the absence of metal salt. According to the Hansen solubility parameters of PEG and PMMA, P4 is more hydrophilic than P2 [38,39]. Thus, the chains of P4 containing the hydrophilic groups were easily located near the water droplets and precipitated around condensed water droplets to prevent their coalescence, which was different from the system of P2 in THF. Consequently, relatively regular pores were generated after evaporation of water droplets using P4/THF.

Second, addition of an appropriate content of metal salt was found to promote the formation of honeycomb films (Figure 6b). In addition, the average pore size can be decreased by increasing metal salt content. This can be explained by the spreading property of the water droplet on the polymer surface. The spreading coefficient S is defined as follows [40]:

S = γ_sg_ − (γ_wg_ + γ_ws_)

where γ_sg_ is the surface tension of the polymer solution; γ_wg_ is the surface tension of the water droplet; and γ_ws_ is the interfacial tension between the polymer solution and the water droplet. The addition of metal salt to the polymer/THF changes the composition of the solution, leading to the increase of γ_sg_. As a result, the water droplets become flatter and lead to the formation of larger ellipsoidal pores on the films. However, the metal salt will transfer from the polymer/THF solution to the water droplets with the further increase of salt content. The presence of metal salt in water droplets increases the surface tension of the water droplets, γ_wg_. Water droplets with a larger surface tension are strongly prone to shrinking their surface area; thus, they can maintain their spheroidal shape. Therefore, with the increase of salt content in the polymer/THF solution, the shape of the water droplets progressively changed from an ellipsoid to a spheroid. Additionally, guided by specific ion effects, the presence of metal ions could affect the solubility of the polymer in the solution. The metal ions are more prone to inducing precipitation of polymer chains depending on their position in the Hofmeister series [41,42]. Therefore, the salting-out effect of metal salt will accelerate the precipitation and solidification of the polymer [43], thereby preventing the growth and condensation of water droplets, which leads to the formation of fewer and smaller pores. Consequently, relatively smaller spherical pores were generated after the evaporation of spherical water droplets.

Last, the addition of excess content of metal salt into the polymer/THF solution will lead to the formation of irregular and larger pores on the films (Figure 6c). During THF evaporation, water droplets condense and are deposited on the solution’s surface. Subsequently, the metal salt will transfer from the polymer/THF solution to the water droplets on a relatively fast timescale, which leads to a significant increase in the surface tension of the water droplets, making the interface unstable. Since metal salt has much more polarity than water, it can cause water droplets to coalesce more strongly and efficiently. If it surpasses the ability of the polymer–Me^n+^ complexes to stabilize the condensed water droplets, the coalescence cannot be prevented due to the high surface tension of the water droplets. Ultimately, disordered and large pores will be observed on the films.

### 3.3. Surface Characterization and Morphology of Metal-Nanoparticle-Functionalized Honeycomb Films

**Figure 7:**
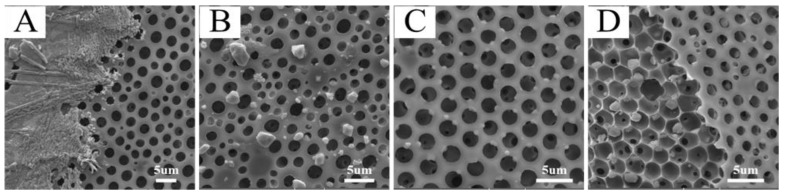
SEM images of honeycomb films with metal salt. (**A**) AgNO_3_ and (**B**) CuCl_2_ on the surface of the film by dropping the metal salt aqueous solution directly on the film. (**C**) The top surface of CuCl_2_-filled honeycomb film. (**D**) CuCl_2_-filled honeycomb film without the top layer.

Generally, the large surface area and high porosity endowed the honeycomb films with superhydrophobicity [2]. Therefore, it was difficult to uniformly distribute metal salt on the surface of honeycomb films by dropping the metal salt aqueous solution directly on the films (Figure 7A,B). On the contrary, the CuCl_2_ was uniformly distributed on the honeycomb films, which were prepared from the CuCl_2_/P4/THF solution. Figure 7C shows the top surface morphology of CuCl_2_-filled honeycomb film; the CuCl_2_ particles were uniformly left in the polymeric cavity and micro-dome. To further confirm that the CuCl_2_ particles were left in the polymeric cavity, the top layer of the honeycomb film was peeled off using an adhesive tape. It is clearly shown that the CuCl_2_ particles were trapped in the holes of the honeycomb film (Figure 7D).

**Figure 8:**
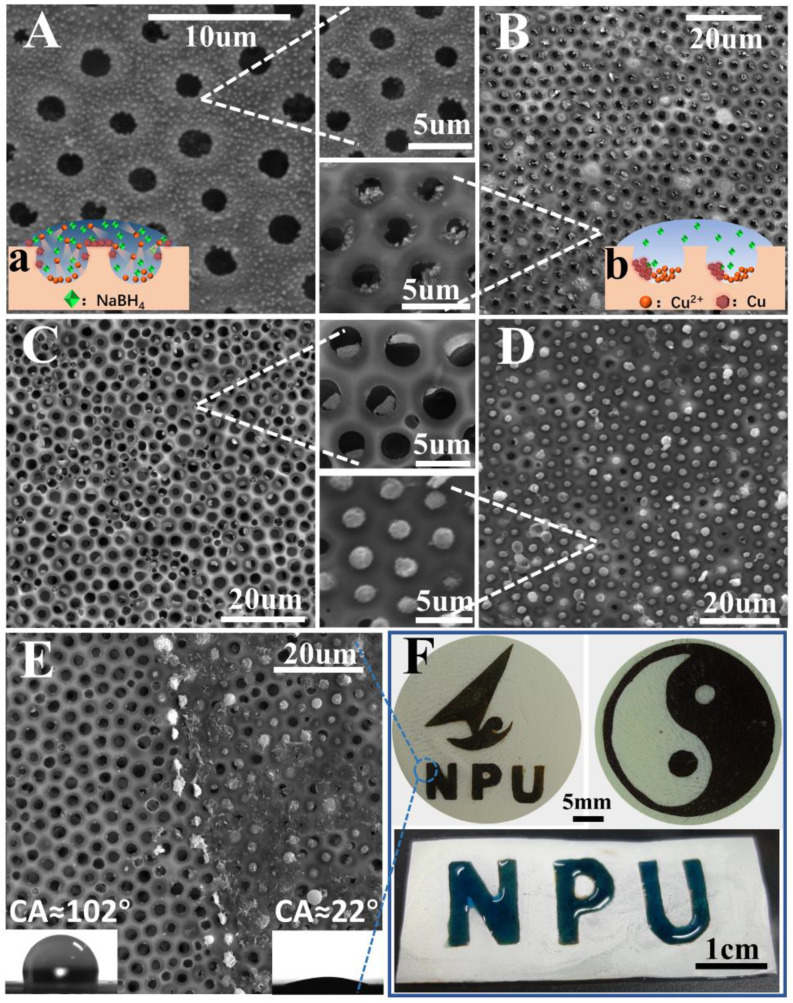
SEM images of honeycomb film with 0.8 wt% CuCl_2_ additions reduced by different concentrations of NaBH_4_: (**A**) 1 mol/L and (**B**) 0.05 mol/L. The film reduced by 0.05 mol/L NaBH_4_ with different additions of CuCl_2_: (**C**) 1.2 wt% and (**D**) 1.6 wt%. A boundary is clearly shown in (**E**). (**F**) Digital photographs of the patterned film filled with Cu nanoparticles; the dark region is filled with Cu nanoparticles. Image illustrating the heart-shaped liquid pattern on the patterned honeycomb film surface with wettability contrast.

Honeycomb films pore-filled with metal nanoparticles were fabricated by using NaBH_4_ to reduce metal salt particles into metal nanoparticles in situ. Notably, the hydrophilicity of the films was enhanced by the presence of metal salt. Therefore, the NaBH_4_ aqueous solution could spread well on the films, and then the metal salt was reduced well. As can be seen from Figure 8B, the honeycomb-patterned porous morphology with Cu-nanoparticle-filled pores was good. The pores and Cu nanoparticles were arranged hexagonally with few defects. More interestingly, the CuCl_2_ particles were initially in the polymeric cavity and were not easily observed from the top surface, while the Cu nanoparticles filled in the polymeric micro-dome and could be easily observed from the top surface. It was noted that the size of the Cu-nanoparticle-filled polymeric micro-dome could be adjusted by simply tuning the content of CuCl_2_. The Cu nanoparticles became larger as CuCl_2_ addition increased (Figure 8B–D).

More interestingly, it was found that metal nanoparticles could be selectively functionalized on the surface or in the pores of the honeycomb films by controlling the concentration of the NaBH_4_. At the higher concentration of NaBH_4_ (1 mol/L), the Cu nanoparticles became smaller and assembled on the surface of the honeycomb films instead of the pores (Figure 8A). However, honeycomb films with Cu-nanoparticle-filled pores were obtained at a lower (0.05 mol/L) concentration of NaBH_4_ (Figure 8B). This phenomenon can be explained as follows [44,45,46,47]: At higher concentrations of NaBH_4_, the reaction was violent and atomic diffusion was high, so the Cu^2+^ could easily diffuse on the surface of film (Figure 8a). The excess reducing agent increased the reduction rate of Cu^2+^ to Cu^0^, which provided a higher number of Cu nuclei. The formation of this higher number of Cu nuclei consumed a large portion of the Cu^2+^, which inhibited the growth of nanocrystals, leading to the formation of small-sized nanoparticles. The resulting small-sized nanoparticles were immobilized on the surface of the film. However, at a lower concentration of NaBH_4_, the reaction was less violent and the atomic diffusion was not high, so the Cu^2+^ could not easily diffuse on the surface of film and remain in the pore (Figure 8b). A slow reduction of the Cu^2+^ was used to prevent the generation of new nuclei, which led to the decreased nuclei numbers. Thus, the Cu nuclei could grow into large Cu nanoparticles in the pores of film.

Furthermore, differently surface-patterned honeycomb films containing Cu nanoparticles in the pores were prepared by using different masks (Figure 8F), which were used to prevent the NaBH_4_ aqueous solution from spreading. Figure 8E shows a clear boundary that formed between the regions functionalized and unfunctionalized by the Cu nanoparticles. The Cu-nanoparticle-functionalized region was hydrophilic (CA ≈ 22°), and the unfunctionalized region was hydrophobic (CA ≈ 102°). As we know, the introduction of Cu nanoparticles will increase the roughness of the surfaces, making the surfaces more hydrophobic. However, too many Cu nanoparticles lead to a decline in the apparent contact angle of water because Cu nanoparticles are hydrophilic [48]. Therefore, between the two factors (i.e., surface roughness and chemistry) that affect the surface wettability, the chemical properties of Cu nanoparticles play a dominant role in this study.

**Figure 9:**
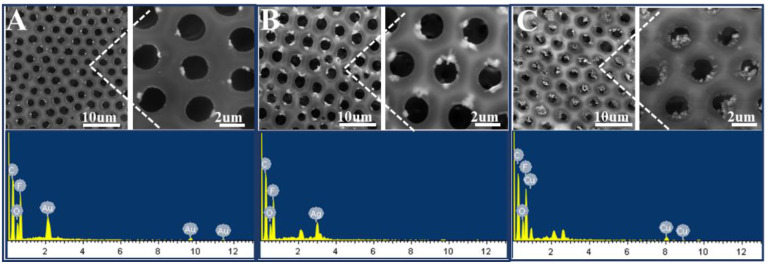
SEM images and corresponding energy-dispersive spectroscopy (EDS) spectra of the honeycomb films filled with metal nanoparticles: Au (**A**), Ag (**B**), and Cu (**C**). The concentrations of metal salt are 0.4 wt% (Au, Ag) and 0.8 wt% (Cu), and they were reduced by 0.05 mol/L NaBH_4_.

**Figure 10:**
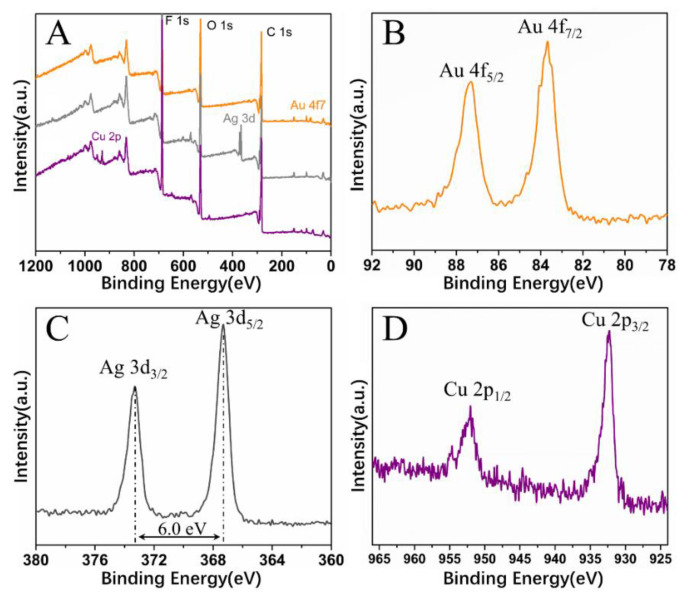
(**A**) X-ray photo-electron spectroscopy (XPS) survey spectra of honeycomb films filled with metal nanoparticles; (**B**–**D**) high-resolution XPS spectra of Au 4f, Ag 3d, and Cu 2p, respectively.

With this proposed strategy, honeycomb-patterned porous films filled with Au or Ag nanoparticles were also fabricated (Figure 9A,B). The EDS spectra support the presence of Au or Ag nanoparticles in the film. In addition, the XPS spectra confirm that metal salts were reduced to metal nanoparticles by NaBH_4_. In the Au4f region (Figure 10B), two peaks at 87.3 and 83.7 eV were assigned to the binding energies of Au(0) 4f_5/2_ and Au(0) 4f_7/2_, respectively. In the Ag3d region (Figure 10C), two peaks at 374.1 and 368.1 eV were assigned to the binding energies of Ag(0) 3d_3/2_ and Ag3d_5/2_, respectively. Similarly, in the Cu2p region (Figure 10D), two peaks at 953.4 and 933.2 eV were assigned to the binding energies of Cu(0) 2p_1/2_ and Cu(0) 2p_3/2_, respectively.

## 4. Conclusions

In summary, honeycomb films with pores selectively filled with metal (Au, Ag, and Cu) nanoparticles were successfully prepared by combining the breath figure method and an in situ reduction reaction. The polymer molecular structures of POSS-based star-shaped polymers played a crucial role in the morphology of the honeycomb films. It was found that the POSS-based star-shaped polymer with relatively more hydrophilic groups made it easy to generate regular honeycomb films. Within a range of conditions, metal salt can promote the formation of regular honeycomb films using the POSS-based star-shaped polymer with relatively more hydrophobic groups. This can be attributed to the formation of polymer–Me^n+^ complexes that could stabilize the condensed water droplets. Metal salt is left in the pores after the evaporation of the solvent and water, which makes it possible to reduce them into metal nanoparticles in situ in the pores. Notably, metal nanoparticles can be selectively reduced in situ in the pores of the honeycomb films using lower (0.05 mol/L) concentrations of NaBH_4_, while they can be assembled on the surface of the honeycomb films at higher (1 mol/L) concentrations of NaBH_4_.

## Figures and Tables

**Table 1:** Sample codes and characteristics of the star-shaped polymers.

Star Polymers	*M*n ^a^ (g/mol)	Repeating Unit Ratio ^b^	Polydispersity Index (PDI ^a^)
P1	1.24 × 10^5^	/	1.14
P2	1.37 × 10^5^	66:42	1.23
P3	7.19 × 10^4^	32:2	1.79
P4	6.00 × 10^4^	35:4	1.20

^a^ Measured by gel permeation chromatography (GPC). ^b^ Calculated according to the *M*n ^a^ and ^1^H nuclear magnetic resonance (NMR) spectra.

## Data Availability

The data presented in this study are available on request from the corresponding author.

## References

[1] Du C., Zhang A., Bai H., Li L. (2013). Robust Microsieves with Excellent Solvent Resistance: Cross-Linkage of Perforated Polymer Films with Honeycomb Structure. ACS Macro Lett..

[2] Li Z., Ma X., Zang D., Hong Q., Guan X. (2015). Honeycomb porous films of pentablock copolymer on liquid substrates via breath figure method and their hydrophobic properties with static and dynamic behaviour. RSC Adv..

[3] Liang J., Ma Y., Sims S., Wu L. (2015). A patterned porous polymer film for localized capture of insulin and glucose-responsive release. J. Mater. Chem. B..

[4] Widawski G., Rawiso M., François B. (1994). Self-organized honeycomb morphology of star-polymer polystyrene films. Nature.

[5] François B., Pitois O., François J. (1995). Polymer films with a self-organized honeycomb morphology. Adv. Mater..

[6] Stenzel M.H., Barner-Kowollik C., Davis T.P. (2006). Formation of honeycomb-structured, porous films via breath figures with different polymer architectures. J. Polym. Sci. Part A Polym. Chem..

[7] Rodríguez-Hernández J., Bormashenko E. (2020). Breath Figures: Mechanisms of Multi-scale Patterning and Strategies for Fabrication and Applications of Microstructured Functional Porous Surfaces.

[8] Yabu H. (2018). Fabrication of honeycomb films by the breath figure technique and their applications. Sci. Technol. Adv. Mater..

[9] Kamei J., Yabu H. (2015). On-Demand Liquid Transportation Using Bioinspired Omniphobic Lubricated Surfaces Based on Self-Organized Honeycomb and Pincushion Films. Adv. Funct. Mater..

[10] Hirai Y., Mayama H., Matsuo Y., Shimomura M. (2017). Uphill Water Transport on a Wettability-Patterned Surface: Experimental and Theoretical Results. ACS Appl. Mater. Interfaces.

[11] Tang P., Hao J. (2010). Directionally electrodeposited gold nanoparticles into honeycomb macropores and their surface-enhanced Raman scattering. New J. Chem..

[12] Dong R., Yan J., Ma H., Fang Y., Hao J. (2011). Dimensional architecture of ferrocenyl-based oligomer honeycomb-patterned films: From monolayer to multilayer. Langmuir.

[13] Liu X., Monzavi T., Gitsov I. (2019). Controlled ATRP Synthesis of Novel Linear-Dendritic Block Copolymers and Their Directed Self-Assembly in Breath Figure Arrays. Polymers.

[14] Chen H.-Y., Liu J.-L., Xu W.-C., Wang Z.-F., Wang C.-Y., Zhang M. (2016). Selective assembly of silver nanoparticles on honeycomb films and their surface-enhanced Raman scattering. Colloids Surf. A.

[15] Dong R., Xu J., Yang Z., Wei G., Zhao W., Yan J., Fang Y., Hao J. (2013). Preparation and functions of hybrid membranes with rings of Ag NPs anchored at the edges of highly ordered honeycomb-patterned pores. Chem. Eur. J..

[16] Nakanishi T., Hirai Y., Kojima M., Yabu H., Shimomura M. (2010). Patterned metallic honeycomb films prepared by photo-patterning and electroless plating. J. Mater. Chem..

[17] Yabu H., Hirai Y., Shimomura M. (2006). Electroless Plating of Honeycomb and Pincushion Polymer Films Prepared by Self-Organization. Langmuir.

[18] Hirai Y., Yabu H., Matsuo Y., Ijiro K., Shimomura M. (2010). Arrays of triangular shaped pincushions for SERS substrates prepared by using self-organization and vapor deposition. Chem. Commun..

[19] Chiang C.-Y., Liu T.-Y., Su Y.-A., Wu C.-H., Cheng Y.-W., Cheng H.-W., Jeng R.-J. (2017). Au Nanoparticles Immobilized on Honeycomb-Like Polymeric Films for Surface-Enhanced Raman Scattering (SERS) Detection. Polymers.

[20] He Y., Chen Y., Xu Q., Xu J., Weng J. (2017). Assembly of Ultrathin Gold Nanowires into Honeycomb Macroporous Pattern Films with High Transparency and Conductivity. ACS Appl. Mater. Interfaces.

[21] Yang P., Huang J., Sun W., Wei Y., Liu Y., Ding L., Bao J., Chen Z.-R. (2016). Exploration of selective decoration of Janus silica particles within polymeric patterned pore arrays. RSC Adv..

[22] Ma H., Gao P., Fan D., Du B., Hao J., Wei Q. (2013). Assembly of graphene nanocomposites into honeycomb-structured macroporous films with enhanced hydrophobicity. New J. Chem..

[23] Kong L., Dong R., Ma H., Hao J. (2013). Au NP Honeycomb-Patterned Films with Controllable Pore Size and Their Surface-Enhanced Raman Scattering. Langmuir.

[24] Ma H., Cui J., Song A., Hao J. (2011). Fabrication of freestanding honeycomb films with through-pore structures via air/water interfacial self-assembly. Chem. Commun..

[25] Jiang X., Zhou X., Zhang Y., Zhang T., Guo Z., Gu N. (2010). Interfacial effects of in situ-synthesized Ag nanoparticles on breath figures. Langmuir.

[26] Li X., Zhang L., Wang Y., Yang X., Zhao N., Zhang X., Xu J. (2011). A bottom-up approach to fabricate patterned surfaces with asymmetrical TiO2 microparticles trapped in the holes of honeycomblike polymer film. J. Am. Chem. Soc..

[27] Shin B.K., Male U., Huh D.S. (2018). In-situ pore filling of TiO_2_ nanoparticles in honeycomb patterned porous films: A modified breath figure method. Polymer.

[28] Pizarro G.d.C., Marambio O.G., Martin-Trasanco R., Sánchez J., Jeria-Orell M., Oyarzún D.P. (2020). Microporous hybrid films from amphiphilic copolymers: Surface coated with ZnS nanoparticles using the breath figure (BF) methodology. Chem. Pap..

[29] Liu Y.-Q., Pan G.-B., Zhang M., Li F. (2013). Micro-patterned composite films with bowl-like SnO_2_ microparticles. Mater. Lett..

[30] Zhang F., Ma Y., Liao J., Breedveld V., Lively R.P. (2018). Solution-Based 3D Printing of Polymers of Intrinsic Microporosity. Macromol. Rapid. Commun..

[31] Naboka O., Sanz-Velasco A., Lundgren P., Enoksson P., Gatenholm P. (2012). Cobalt (II) chloride promoted formation of honeycomb patterned cellulose acetate films. J. Colloid Interface Sci..

[32] Chen Y.-T., Tseng C.-C., Tsai H.-J., Hsu W.-K. (2019). Humidity controlled light transmittance of salt modified porous polymeric membranes. Chem. Phys. Lett..

[33] Mir S.H., Ochiai B. (2016). Fabrication of Polymer-Ag Honeycomb Hybrid Film by Metal Complexation Induced Phase Separation at the Air/Water Interface. Macromol. Mater. Eng..

[34] Uyen Thi P.N., Male U., Huh D.S. (2018). In situ surface selective functionalization of honeycomb patterned porous poly(ε-caprolactone) films using reactive substrate. Polymer.

[35] Qiang X., Ma X., Li Z., Hou X. (2014). Synthesis of star-shaped polyhedral oligomeric silsesquioxane (POSS) fluorinated acrylates for hydrophobic honeycomb porous film application. Colloid Polym. Sci..

[36] Hong Q., Ma X., Li Z., Chen F., Zhang Q. (2016). Tuning the surface hydrophobicity of honeycomb porous films fabricated by star-shaped POSS-fluorinated acrylates polymer via breath-figure-templated self-assembly. Mater. Des..

[37] Wu B.-H., Zhu L.-W., Ou Y., Tang W., Wan L.-S., Xu Z.-K. (2015). Systematic investigation on the formation of honeycomb-patterned porous films from amphiphilic block copolymers. J. Phys. Chem. C..

[38] Yune P.S., Kilduff J.E., Belfort G. (2011). Using co-solvents and high throughput to maximize protein resistance for poly(ethylene glycol)-grafted poly(ether sulfone) UF membranes. J. Membr. Sci..

[39] Patra N., Barone A.C., Salerno M. (2011). Solvent effects on the thermal and mechanical properties of poly(methyl methacrylate) casted from concentrated solutions. Adv. Polym. Technol..

[40] Dobbs H., Bonn D.J.L. (2001). Predicting Wetting Behavior from Initial Spreading Coefficients. Langmuir.

[41] Hofmeister F. (1888). Zur lehre von der wirkung der salze. Arch. Exp. Pathol. Pharmakol..

[42] Moghaddam S.Z., Thormann E. (2019). The Hofmeister series: Specific ion effects in aqueous polymer solutions. J. Colloid Interface Sci..

[43] Zhang S.Y., Miao H., Zhang H.M., Zhou J.H., Zhuang Q., Zeng Y.J., Gao Z., Yuan J., Sun J.K. (2020). Accelerating Crystallization of Open Organic Materials by Poly (ionic liquid)s. Angew. Chem..

[44] LaMer V.K., Dinegar R.H. (1950). Theory, production and mechanism of formation of monodispersed hydrosols. J. Am. Chem. Soc..

[45] Baeza J., Calvo L., Gilarranz M., Mohedano A., Casas J., Rodriguez J. (2012). Catalytic behavior of size-controlled palladium nanoparticles in the hydrodechlorination of 4-chlorophenol in aqueous phase. J. Catal..

[46] Teranishi T., Miyake M. (1998). Size control of palladium nanoparticles and their crystal structures. Chem. Mater..

[47] Yung K.C., Law C.M.T., Lee C.P., Cheung B., Yue T.M. (2011). Size Control and Characterization of Sn-Ag-Cu Lead-Free Nanosolders by a Chemical Reduction Process. J. Electron. Mater..

[48] Zhong Q.Z., Yi M.H., Du Y., He A., Xu Z.K., Wan L.S. (2017). Multiple Liquid Manipulations on Patterned Surfaces with Tunable Adhesive Property. Adv. Mater. Interfaces.

